# HLA and killer cell immunoglobulin-like receptor (KIRs) genotyping in patients with acute ischemic stroke

**DOI:** 10.1186/s12974-019-1469-5

**Published:** 2019-04-17

**Authors:** Antonino Tuttolomondo, Domenico Di Raimondo, Rosaria Pecoraro, Alessandra Casuccio, Danilo Di Bona, Anna Aiello, Giulia Accardi, Valentina Arnao, Giuseppe Clemente, Vittoriano Della Corte, Carlo Maida, Irene Simonetta, Calogero Caruso, Rosario Squatrito, Antonio Pinto, on behalf of KIRIIND (KIR Infectious and Inflammatory Diseases) Collaborative Group, Antonino Tuttolomondo, Domenico Di Raimondo, Danilo Di Bona, Calogero Caruso, Antonio Pinto

**Affiliations:** 1 Internal Medicine and Stroke Care Ward, Department of Health Promotion Sciences, Maternal and Infant Care, Internal Medicine and Medical Specialties (PROMISE), University of Palermo, P.zza delle Cliniche n.2, 90127 Palermo, Italy; 2 Department of Health Promotion Sciences, Maternal and Infant Care, Internal Medicine and Medical Specialties (PROMISE), University of Palermo, P.zza delle Cliniche n.2, 90127 Palermo, Italy; 3 Dipartimento di Biopatologia e Biotecnologie Mediche, Universita’ degli Studi di Palermo, Palermo, Italy; 4 Dipartimento di BioMedicina Sperimentale e Neuroscienze Cliniche, Università degli Studi di Palermo, Palermo, Italy; 5 School and Chair of Allergology, Dipartimento delle Emergenze e Trapianti d’Organo, University of Bari, Bari, Italy; 6 Pronto Soccorso Unit, Giuseppe Giglio Hospital, Cefalù, Italy; 7 PhD Programme in Clinical Medicine and Behavioural Sciences, University of Palermo, Palermo, PA 90133 Italy; 8 PhD Programme in Molecular and Clinical Medicine, University of Palermo, Palermo, PA 90133 Italy

**Keywords:** Stroke, Killer immunoglobulin-like receptors (KIRs), HLA

## Abstract

**Introduction:**

In humans, a major component of natural killer (NK) and T cell target recognition depends on the surveillance of human leukocyte antigen (HLA) class I molecules by killer immunoglobulin-like receptors (KIRs).

**Aims:**

To implement the knowledge about the immunological genetic background of acute ischemic stroke susceptibility in relation to the frequency of the KIR genes and HLA alleles.

**Methods:**

Subjects with acute ischemic stroke and subjects without stroke were genotyped for the presence of KIR genes and of the three major KIR ligand groups, HLA-C1, HLA-C2, and HLA-Bw4, both HLA-B and HLA-A loci.

**Results:**

Between November 2013 and February 2016, consecutive patients with acute ischemic stroke were recruited. As healthy controls, we enrolled subjects without acute ischemic stroke. Subjects with acute ischemic stroke in comparison with controls showed a higher frequency of 2DL3, 2DL5B, 2DS2, and 2DS4 KIR genes and a lower frequency of HLA-B-Bw4^I^ alleles. Subjects without acute ischemic stroke showed a higher frequency of interaction between KIR 2DS2 and HLAC2. We also observed a higher frequency of 2DL3 and 2 DL4 KIR genes in subjects with atherosclerotic (LAAS) subtype. Multiple logistic regression analysis showed a protective effect towards stroke of HLA-B-Bw4^I^ and interaction between KIR 2DL2 and HLAC1 and 2DS2-HLAC2 and a detrimental effect of 2DL2-HLA-C1_A interactions.

**Conclusion:**

Our findings of a higher frequency of activating KIR genes seem to be consistent with findings previously reported patients with coronary syndrome. This higher frequency of “proinflammatory” genes in subjects with ischemic stroke could also explain the immunoinflammatory activation of the acute phase of stroke.

## Background and purpose

Evidence for the contribution of T cell-mediated immunity to cardiovascular diseases has only recently emerged [1, 2]. T cells infiltrate atherosclerotic plaques, and in these sites, proinflammatory immune responses mediated by CD4^+^ T-helper type 1 (T_H_1) and CD8^+^ cytotoxic T lymphocytes are preponderant [1–4]. Natural killer (NK) cells activating or inhibition pathways mediated by human leukocyte antigen (HLA) expression could have a potential detrimental or protective role towards inflammation-mediated acute complications of atherosclerosis such as acute ischemic stroke.

In humans, a major component of NK and T cell target recognition depends on the surveillance of human leukocyte antigen (HLA) class I molecules by killer immunoglobulin-like receptors (KIRs), a family of diverse activating or inhibitory receptors that are expressed on the surface of NK cells and T cell subsets [5]. Different KIRs can transmit inhibitory or activating signals to the cell, and combinations of KIR genes synergize to generate genes with widely differing balance between activating and inhibitory types.

KIR receptors have been reported as involved in the susceptibility to several diseases, including celiac disease, rheumatoid arthritis, systemic lupus erythematosus, infectious diseases, and cancer [4, 5]. Our group previously reported [8] that immunocompetent subjects carrying the homozygous A haplotype or the HLABw4^T^ allele are at higher risk of developing symptomatic disease after primary cytomegalovirus (CMV) infection.

Although HLA genes, as well as the innate immune genes KIR, are considered to be important determinants of T cell involvement in inflammation, few studies, to our knowledge, evaluated their role in the clinical setting of susceptibility of acute complications of atherosclerotic disease [5, 6].

One study [7] reported that CD4+CD28^null^ T cell clones from patients with acute coronary syndrome (ACS) expressed a broader spectrum of KIRs with preference for the stimulatory variant, CD158j, and that CD4+ T cell clones from patients acquired de novo expression of the DAP12 molecule, an adapter chain that transmits CD158j-derived signals. Consequently, these T cells acquire cytolytic capability that may suggest T cell involvement also in acute complication of atherosclerosis also indicating a possible role of KIR genetic background in regulation of inflammatory cell involvement in acute cardiovascular event.

No study, to the best of our knowledge, analyzed the relationship between KIR aplotype and HLA allele and the risk of developing an acute ischemic stroke.

### Study hypothesis

- The principal hypothesis of our study was that a higher risk of developing an ischemic stroke may be related to a higher frequency of some KIR genes and their HLA ligands and of their interactions in comparison with subjects without ischemic stroke.
- An additional hypothesis was that a higher risk of developing certain subtypes of ischemic stroke may be due to a higher frequency of some genetic variants of KIR and their HLA ligands or of their interactions.

### Aim of the study

The aim of this analytical cross-sectional study was:

- To assess if genetic variants of KIR and their HLA ligands may influence susceptibility to ischemic stroke and its TOAST subtype and to implement the knowledge about the immunological and genetic background of acute ischemic stroke susceptibility in connection with the expression of the KIR and HLA alleles
- To further characterize immune-inflammatory degree of the acute phase of ischemic stroke by means evaluation of peripheral frequency of CD28 ^null^ cells in a sample of patients with acute stroke in comparison with subjects without ischemic stroke

## Materials and methods

Between November 2013 and July 2016, consecutive patients with acute ischemic stroke were recruited from Internal Medicine with Stroke Care ward of Policlinico “P. Giaccone”, University of Palermo and Pronto Soccorso Unit of Giuseppe Giglia Hospital, Cefalù (Palermo). As controls, we enrolled consecutive healthy subjects admitted to our ward in the same recruitment period and without acute ischemic stroke and without exclusion criteria (see above).

Ischemic stroke has been defined as: “An episode of neurological dysfunction caused by focal cerebral, spinal, or retinal infarction lasting more than 24 hours” [8].

For stroke patients, we evaluated medical history, 12-lead ECG, 24-h electrocardiography monitoring, trans-thoracic echocardiography, carotid ultrasound, brain CT, or MRI at admission.

Patients and controls were excluded if they had one of these exclusion criteria: rheumatologic disorders, chronic inflammatory disease, acute systemic infections, recent venous thrombosis, recent acute myocardial infarction (AMI) (within 3 months), and recent cerebrovascular event (TIA or stroke within 6 months)*.*

Type 2 diabetes mellitus was determined using a clinically based algorithm that considered age at onset, presenting weight and symptoms, family history, onset of insulin treatment, and history of ketoacidosis [9]. Hypertension was defined according to the 2013 ESH/ESC guidelines [10]. Hypercholesterolemia was defined as total serum cholesterol ≥ 200 mg/dL, and hypertriglyceridemia as total serum triglyceride ≥ 150 mg/dL on the basis of the National Cholesterol Education Program–Adult Treatment Panel III reports [11, 12]. Coronary artery disease has been evaluated by means the presence of clinical symptomatology of angina, myocardial infarction, or any previous coronary revascularization.

Cerebrovascular disease (TIA/ischemic stroke) has been evaluated by means patient history, neurological exam, and hospital records.

### Ethical approval and consent to participate

This protocol study was approved by the Ethics Committee of the Policlinico P. Giaccone Hospital and of Giuseppe Giglia Hospital, Cefalù (Palermo). All patients gave their written informed consent to participate in the study, as well as for sampling and banking of the biological material.

### Stroke subtype evaluation

The type of acute ischemic stroke was classified according to the TOAST classification [13]: (1) large artery atherosclerosis (LAAS), (2) cardioembolic infarct (CEI), (3) lacunar infarct (LAC), (4) stroke of other determined etiology (ODE), and (5) stroke of undetermined etiology (UDE).

### Cell isolation, staining, and flow cytometry

Peripheral blood has been drawn at 48 h after symptom onset and after informed consent had been obtained from the patient or his/her authorized representative. Peripheral blood mononuclear cells (PBMCs) have been obtained by density gradient centrifugation using the lymphocyte separation medium (ICN Pharmaceutical, Costa Mesa, CA). This protocol yields an average PBMC composition of 60% T cells, 15% monocytes/macrophages, 10% B cells, and 15% natural killer cells [29]. White cells were obtained from 2 ml of peripheral EDTA-anticoagulated venous blood. Cells were labeled with human monoclonal anti-CD4 antibodies conjugated with fluorescein isothiocyanate and anti-CD28 antibodies conjugated with phycoerythrine (Becton Dickinson). Mouse IgG1 antibodies conjugated with fluorescein isothiocyanate (IgG1-FITC) and IgG2a conjugated with phycoerythrine IgG2a-PE (Becton Dickinson) were used as the isotype controls. The samples were incubated for 30 min in the dark at ambient temperature, washed with 5% saline, and centrifuged. The pellet was suspended in 1% formalin. Expression of the CD28 receptor on lymphocytes was studied with a FACSCalibur flow cytometer (FACSCalibur/Sysmex XT1800i dual platform) operating with CellQuest OS2 software. The population of CD4+CD28^null^ was expressed as a percentage of CD4+ cells (CD4+CD28^null^ and CD4+CD28+).

### HLA and KIR genotyping

Peripheral whole blood samples were collected as indicated, and genomic DNA was extracted from leukocytes by a commercial kit (PureLink® Genomic DNA, ThermoFisher Scientific, Waltham, MA, USA). Using the polymerase chain reaction sequence-specific primer (PCR-SSP) technique, the DNA subjects with acute ischemic stroke and subjects without stroke were genotyped for the presence of the three major KIR ligand groups, HLA-C1, HLA-C2, and HLA-Bw4, both HLA-B and HLA-A loci (Epitop-TYPE kit; BAG Health Care GmbH, Lich, Germany). HLA-C and Bw4 KIR ligand groups were assigned directly by using specific oligonucleotide primers to type the codon corresponding to amino acid 80 for HLA-C (HLA-C1, Cw alleles with asparagine at position 80; HLA-C2, Cw alleles with lysine at position 80) and for HLA-Bw4 (Bw4-I, Bw alleles with isoleucine at position 80; Bw4-T, Bw alleles with threonine at position 80). KIR genotyping was performed for both inhibitory and activating KIR using the KIR-TYPE kit (BAG Health Care GmbH).

The following KIR genes were analyzed, which included the inhibitory receptors KIR2DL1, 2DL2, 2DL3, 2DL5, 3DL1, 3DL2, and 3DL3; the activating receptors 2DL4, 2DS1, 2DS2, 2DS3, 2DS4, 2DS5, and 3DS1; two pseudogenes (2DP1 and 3DP1) and the common variants of KIR2DL5 (KIR2DL5A, KIR2DL5B); the KIR2DS4 alleles; and KIR3DP1 alleles. KIR gene profiles were determined by the presence or absence of each KIR gene.

The detection of individual receptors Kir/HLA ligands was performed with the sequence-specific primer method. Therefore, the amplification occurs only if the primers are complementary to the target sequence and is then highlighted to agarose gel electrophoresis. The specific primers were selected so as to detect single KIR/HLA ligand genes.

### Statistical analysis

Statistical analysis of quantitative and qualitative data, including descriptive statistics, was performed for all items. Continuous data are expressed as mean ± SD, unless otherwise specified. Baseline differences between groups were assessed by the chi-square test or Fisher exact test, as needed for categorical variables, and by the independent Student *t* test for continuous parameters if the data were normally distributed. If the data did not show a normal distribution, a non-parametric test was used (Mann Whitney’s *U* test). Logistic regression analysis examined the correlation between patient characteristics (independent variables) and patient groups (dependent variable) in simple and multiple regression models. An overall sample size of 124 patients (62 for each study group) has been calculated to document a difference of at least 20% in the prevalence of KIR genes and HLA alleles between the patient groups. The sample sizes took into account a significance level of 5% and a power of 80%.

Data were analyzed by IBM SPSS Software 22 version (IBM Corp., Armonk, NY, USA). All *p* values were two-sided, and *p* < 0.05 was considered statistically significant.

## Results

We recruited 149 subjects with acute ischemic stroke. We subsequently excluded 33 patients with incomplete data and 10 patients for exclusion criteria. In the end, data from 116 patients with acute ischemic stroke and 66 subjects without acute ischemic stroke were analyzed.

General and clinical characteristics of patients with acute ischemic stroke and of subjects without acute ischemic stroke are listed in Table 1.

**Table 1:** General, demographic, and clinical findings in subjects with acute ischemic stroke and in subjects without ischemic stroke

Variables	Subjects with acute ischemic stroke (*n* 116)	Subjects without acute ischemic stroke (*n* 66)	*p*
Sex (M/F) (*n*)	59/57	29/37	0.43
Age (years) (mean ± ds)	75.5 ± 11.6	73.7 ± 10.7	0.28
Diabetes (*n*/%)	48 (41.37)	30 (45.4)	0.62
Hypertension (*n*/%)	103 (87.9)	53 (80)	0.12
Hypercholesterolaemia (*n*/%)	41 (35.34)	27 (40.90)	0.51
Hypertriclyceridaemia (*n*/%)	26 (22.41)	19 (28.78)	0.32
Atrial fibrillation (*n*/%)	53 (45.68)	28 (42.46)	0.67
Previous stroke (*n*/%)	56 (48.27)	12 (18.18)	< 0.0001
Glucose blood levels (mmol/L)	7.99 ± 3.27	6.05 ± 1.91	< 0.0001
SBP (mm/Hg) (mean ± ds)	15 l.4 ± 27.4	132.1 ± 11.4	< 0.0001
DBP (mm/Hg) (mean ± ds)	82.05 ± 14.43	78.8 ± 6.6	0.094
WBC (mean ± ds)	9448.7 ± 4978.17	7208.7 ± 2141.6	< 0.0001
Neutrophil (%) (mean ± ds)	72.3 ± 10.7	61.87 ± 9.5	0.32
ESR (mm/h) (mean ± ds)	29.07 ± 17.5	14.7 ± 9.16	< 0.0001
CRP (mg/dL) (mean ± ds)	3.84 ± 5.56	1.7 ± 1.6	0.004
TOAST subtype			
LAAS	45 (38.8)		
Lacunar	27 (23.3)		
CEI	40 (34.5)		
ODE	4 (3.4)		
NIHSS (mean ± sd)			
SSSS (mean ± sd)			
CD4+ cells (%) (mean ± sd)	50.21 ± 8.31	34.12 ± 6.81	< 0.0001
CD4+CD28− (%) (mean ± sd)	5.70 ± 2.33	2.78 ± 0.93	< 0.0001
TNF-α (pg/ml) (mean ± sd)	18.7 ± 3.28	12.34 ± 4.54	0.035
IL-6 (pg/ml) (mean ± sd)	22.10 ± 12.21	4.22 ± 1.44	< 0.0001

*SBP* systolic blood pressure, *DBP* diastolic blood pressure, *WBC* white blood cell, *ESR* erythrocyte sedimentation rate, *CRP* C-reactive protein, *LAAS* large artery atherosclerotic stroke, *CEI* cardioembolic infarct, *ODE* other determined etiology, *NIHSS* National Institutes of Health Stroke Scale, *SSS* Scandinavian Stroke Scale Score, *CD4+* cluster of differentiation 4, *CD28−* cluster of differentiation 28 null cell, *TNF-α* tumor necrosis factor alfa, *IL-6* interleukin-6

The mean age of patients with acute ischemic stroke was 75.5 ± 11.6 years in subjects with stroke and 73.7 ± 10.7 years in subjects without acute ischemic stroke.

Subjects with acute ischemic stroke showed in comparison with subjects without stroke a similar prevalence of hypertension, diabetes, hypercholesterolaemia, and atrial fibrillation, whereas subjects with ischemic stroke showed in comparison with subjects without acute stroke a higher prevalence of previous stroke (48.3% vs 18.2%; *p* < 0.0001) and higher mean glucose blood levels (7.99 ± 3.27 mg/dl vs 6.05 ± 1.91 mg/dl; *p* < 0.0001), higher mean SBP values (151.4 ± 27.4 mm/Hg vs 132.1 ± 11.4 mm/Hg; *p* < 0.0001), higher SBP values (15 l.4 ± 27.4 vs 132.1 ± 11.4/Hg; *p* < 0.0001), higher mean WBC count (9448.7 ± 4978.17 vs 7208.7 ± 2141.6; *p* < 0.0001), and higher ESR (29.07 ± 17.5 mm/h vs 14.7 ± 9.16; *p* < 0.0001) and CRP (3.84 ± 5.56 vs 1.7 ± 1.6; *p* = 0.004) mean values.

Subjects with acute ischemic stroke showed in comparison with subjects without ischemic stroke higher peripheral percentage of CD4+ (50.21 ± 8.31% vs 34.12 ± 6.81%; *p* < 0.0001) and CD28^null^ (5.70 ± 2.33% vs 2.78 ± 0.93%; *p* < 0.0001) cells; furthermore, subjects with stroke showed higher serum levels of TNF-α (18.7 ± 3.28 pg/ml vs 12.34 ± 4.54 pg/ml; *p* = 0.035) and IL-6 (22.10 ± 12.21 pg/ml vs 4.22 ± 1.44 pg/ml; *p* < 0.0001) (see Table 1).

**Fig. 1:**
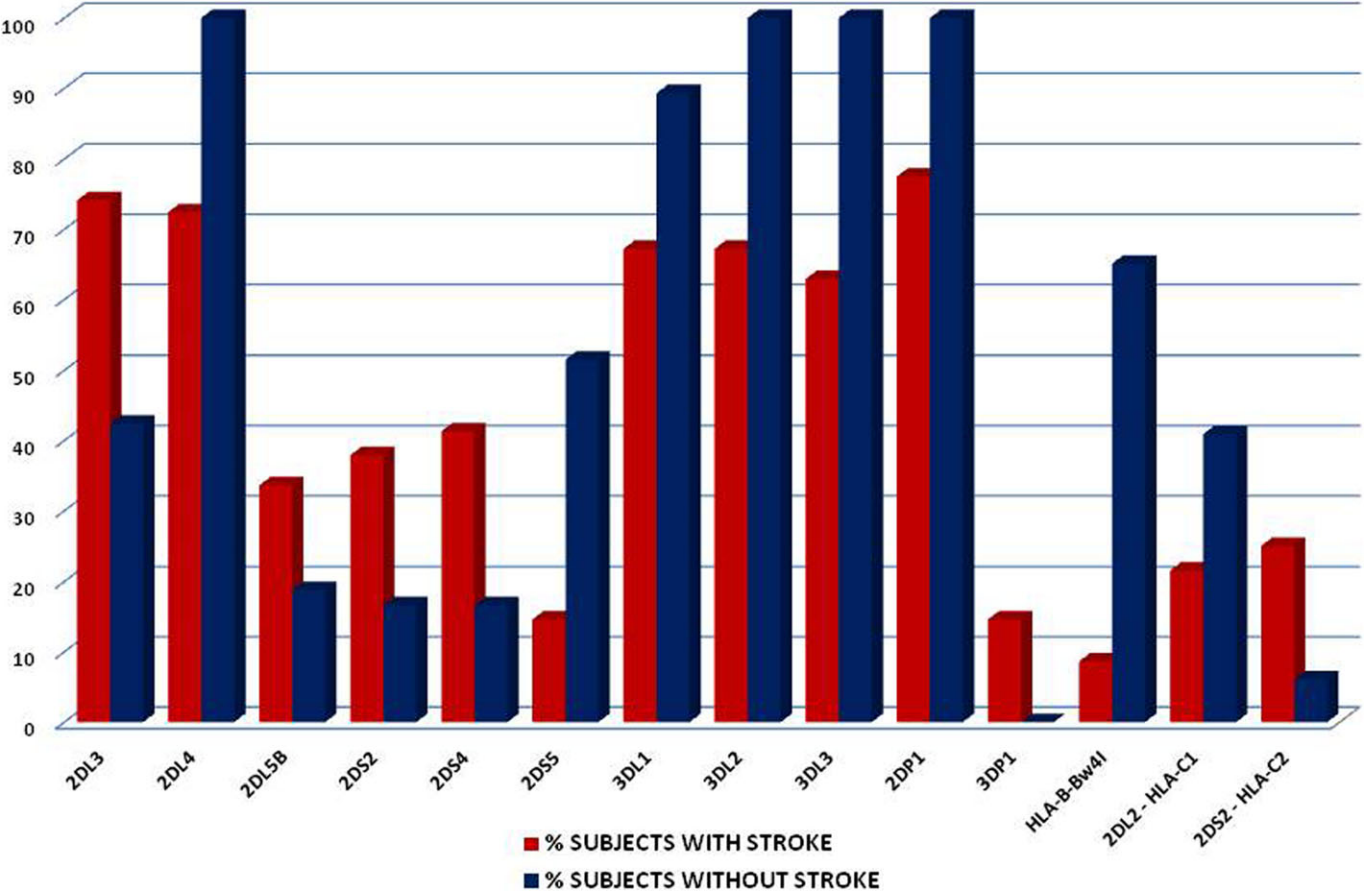
frequency of KIR genes and of interaction KIR-HLA in subjects with ischemic stroke

KIR genotype analysis of patients with acute ischemic stroke in comparison with subjects without ischemic stroke showed a higher frequency of 2DL3 (74.1% vs 42.4% *p* < 0.0001), 2DL5B (33.6% vs 18.8%; *p* = 0.03), 2DS2 (37.9% vs 16.6%; *p* = 0.003), 2DS4 (41.3 vs 16.6; *p* < 0.0001), and 3DP1 (14.6% vs 0; *p* < 0.0001) KIR genes (see Table 2 and Fig. 1) and a lower frequency of 3DL1 (67.2% vs 89.3%; *p* < 0.0001), 3DL2 (67.2% vs 100%; *p* < 0.0001), 3DL3 (62.9% vs 100%; *p* < 0.0001), 2DS5 (14.6% vs 51.5%; *p* < 0.0001), and 2DP1 (77.5% vs 100%) KIR genes in comparison with subjects without stroke. No difference was reported in the frequency of KIR haplotypes between the groups.

**Table 2:** Frequencies of KIR haplotypes and HLA alleles among individuals acute ischemic stroke and in subjects without stroke

Aplotipo KIR	Subjects with acute ischemic stroke (*n* 116)	Subjects without acute ischemic stroke (*n* 66)	*p*
2DL1 (*n*/%)	64 (55.17)	43 (65.16)	0.21
2DL2 (*n*/%)	41 (35.34)	26 (39.3)	0.63
2DL3 (*n*/%)	86 (74.1)	28 (42.4)	< 0.0001
2DL4 (*n*/%)	84 (72.4)	66 (100)	< 0.0001
2DL5A (*n*/%)	14 (12.06)	12 (18.8)	0.27
2DL5B (*n*/%)	39 (33.6)	12 (18.8)	0.03
2DS1 (*n*/%)	24 (20.6)	18 (27.2)	0.36
2DS2 (*n*/%)	44 (37.9)	11 (16.6)	0.003
2DS3 (*n*/%)	31 (26.7)	17 (25.7)	1
2DS4 (*n*/%)	48 (41.3)	11 (16.6)	0.001
2DS5 (*n*/%)	17 (14.6)	34 (51.5)	< 0.0001
3DL1 (*n*/%)	78 (67.2)	59 (89.3)	0.001
3DL2 (*n*/%)	78 (67.2)	66 (100)	< 0.0001
3DL3 (*n*/%)	73 (62.9)	66 (100)	< 0.0001
3DS1 (*n*/%)	60 (51.7)	37 (56.06)	0.64
2DP1 (*n*/%)	90 (77.5)	66 (100)	< 0.0001
3DP1 (*n*/%)	17 (14.6)	0	< 0.0001
3DP1*003 (*n*/%)	73 (62.9)	66 (100)	< 0.0001
KIR-aplotypes			
HLA-A (*n*/%)	52 (44.8)	30 (45.4)	1.0
HLA-A+B (*n*/%)	62 (53.4)	37 (56.0)	0.76
HLA alleles			
HLA-Bw4 T (*n*/%)	31 (26.7)	13 (19.6)	0.36
HLA-A-BW4 (*n*/%)	31 (26.7)	16 (24.2)	0.85
HLA-B-Bw4^I^ (*n*/%)	10 (8.6)	43 (65.1)	< 0.0001
HLA-C1 (*n*/%)	38 (32.7)	26 (39.3)	0.420
HLA-C2 (*n*/%)	36 (31.03)	19 (28.7)	0.86

*KIR* killer cell immunoglobulin-like receptor, *HLA* human leukocyte antigen

With regard to HLA alleles, subjects without ischemic stroke showed in comparison with stroke subjects a significantly higher frequency of HLA-B-Bw4^I^ alleles (65.1% vs 8.6%; *p* < 0.0001) (see Table 2 and Fig. 1).

With regard to KIR-HLA ligand interactions, subjects with acute ischemic stroke showed in comparison with subjects without acute stroke a higher frequency of interaction between KIR2DS2 and HLAC2 (25% vs 6.06%; *p* = 0.001) (see Table 3 and Fig. 1) and a lower frequency of interaction between KIR2DL2 and HLA-C1 (21.55% vs 40.9%; *p* = 0.007).

**Table 3:** Frequencies of KIR-HLA combinations among individuals with acute ischemic stroke and in subjects without stroke

HLA and KIR interaction	Subjects with acute ischemic stroke	Subjects without acute ischemic stroke	*p*
2DL2-HLA-C1 (*n*/%)	25 (21.55)	27 (40.9)	0.007
2DL3-HLA-C1 (*n*/%)	38 (32.75)	22 (33.3)	0.93
2DS2-HLA-C1 (*n*/%)	37 (31.89)	23 (34.48)	0.94
2DS2-HLA-C2 (*n*/%)	29 (25.0)	4 (6.06)	0.001
2DL2-HLA-C2 (*n*/%)	26 (22.41)	11 (16.67)	0.34
2DS1-HLA-C2 (*n*/%)	15 (12.93)	5 (7.57)	0.42
2DL1-HLA-C1 (*n*/%)	16 (13.79)	7 (10.6)	0.52
2DL1-HLA-C2 (*n*/%)	17 (14.65)	13 (13.79)	0.41

*KIR* killer cell immunoglobulin-like receptor, *HLA* human leukocyte antigen

Multiple logistic regression analysis considering variables predictive of ischemic stroke showed the protective effect of HLA-B-Bw4^I^ alleles (*B* = − 3.649; *p* < 0.0001) and of interaction between KIR2DL2 and HLA-C1 (*B* = − 1.901; *p* = 0.004), whereas a detrimental effect was observed with regard to interaction between KIR2DS2 and HLAC2 (*B* = 2.687; *p* = 0.004) and between KIR2DL2 and HLA-C1_A (*B* = 1.847; *p* = 0.014) (see Table 4).

**Table 4:** Logistic regression model to predict the presence of acute ischemic stroke in relation to prevalence of KIR haplotypes

Variable	Beta	Exp(B)	95% confidence interval for Exp(B)	*p* value
HLA-A-Bw4	0.16	1.18	0.42–3.26	0.745
HLA-A aplotype	0.17	1.19	0.48–2.9	0.702
HLA-C1	0.09	1.09	0.35–3.4	0.874
HLA-C2	0.55	1.73	0.53–5.69	0.361
HLA-B-Bw4^T^	0.30	1.35	0.44–4.11	0.593
HLA-B-Bw4^I^	− 3.64	0.02	0.008–0.08	< 0.0001
2DL2-HLA-C1	− 1.90	0.149	0.041–0.55	0.004
2DL3-HLA-C1	0.32	1.37	0.50–3.75	0.529
2DS2-HLA-C1	0.68	1.97	0.67–5.83	0.216
2DS2-HLA-C2	2.68	14.68	2.33–92.4	0.004
2DL2-HLA-C2	0.13	1.14	0.34–3.84	0.825
2DS1-HLA-C2	− 0.27	0.75	0.21–2.64	0.667
2DL1-HLA-C1	0.37	1.45	0.34–6.07	0.607
2DL2-HLA-C1_A	1.84	6.33	1.46–27.47	0.014
2DL1-HLA-C2	0.03	1.03	0.30–3.59	0.953

*KIR* killer cell immunoglobulin-like receptor, *HLA* human leukocyte antigen

General, demographic, and clinical findings in subjects with acute ischemic stroke in relation to TOAST subtype are listed in Table 5.

**Table 5:** General, demographic, and clinical findings of KIR haplotype, HLA allele, and their combination frequencies in subjects with acute ischemic stroke in relation to TOAST subtype

Variables	LAAS (*n* 45)	Lacunar (27)	Cardioembolic (40)	*p*
Male (*n*/%)	29 (64.4)	12 (44.4)	15 (37.5)	0.057
Hypertension (*n*/%)	39 (86.6)	24 (88.8)	36 (90)	0.85
Diabetes (*n*/%)	20 (44.4)	13 (48.14)	15 (37.5)	0.40
Hypercholesterolaemia (*n*/%)	24 (53.3)	6 (22.22)	10 (25)	0.011
Hypertriglyceridaemia (*n*/%)	12 (26.66)	5 (18.51)	9 (22.5)	0.84
Atrial fibrillation (*n*/%)	8 (17.77)	8 (29.62)	35 (87.5)	0.0001
Previous stroke (*n*/%)	21 (46.66)	13 (48.14)	21 (52.5)	0.90
2DL1 (*n*/%)	27 (60)	13 (48.14)	22 (55)	0.75
2DL2 (*n*/%)	20 (44.4)	7 (25.92)	12 (30)	0.20
2DL3 (*n*/%)	39 (86.6)	17 (62.96)	29 (72.5)	0.033
2DL4 (*n*/%)	40 (88.8)	16 (59.25)	26 (65)	0.010
2DL5A (*n*/%)	6 (13.33)	1 (3.73)	7 (17.5)	0.37
2DL5B (*n*/%)	13 (28.8)	11 (40.7)	14 (35)	0.76
2DS1 (*n*/%)	7 (15.5)	7 (25.9)	9 (22.5)	0.52
2DS2 (*n*/%)	14 (31.1)	12 (44.4)	17 (42.5)	0.61
2DS3 (*n*/%)	13 (28.8)	6 (22.2)	10 (25)	*0.92*
2DS4 (*n*/%)	20 (44.4)	10 (37.03)	16 (40)	0.90
2DS5 (*n*/%)	5 (11.11)	4 (14.81)	8 (20)	0.73
3DL1 (*n*/%)	34 (75.55)	13 (48.14)	28 (70)	0.073
3DL2 (*n*/%)	37 (82.22)	14 (51.85)	25 (62.5)	0.016
3DL3 (*n*/%)	36 (80)	13 (48.14)	22 (55)	0.015
3DS1 (*n*/%)	23 (51.1)	14 (51.85)	23 (57.5)	0.35
2DP1 (*n*/%)	39 (86.6)	19 (70.37)	30 (75)	0.26
3DP1 (*n*/%)	3 (6.66)	4 (14.81)	10 (25)	0.11
HLA-A-Bw4 (*n*/%)	8 (17.77)	9 (33.3)	12 (30)	0.33
HLA-A haplotype (*n*/%)	18 (40)	12 (44.4)	20 (50)	0.68
HLA-C1 haplotype (*n*/%)	13 (28.88)	5 (18.51)	19 (47.5)	0.069
HLA-C2 haplotype (*n*/%)	12 (26.66)	9 (33.3)	14 (35)	0.83
HLA-B-Bw4-^T^ (*n*/%)	14 (31.11)	4 (14.81)	12 (30)	0.37
HLA-B-Bw4 ^I^ (*n*/%)	3 (6.66)	1 (3.7)	6 (15)	0.42
2DL2-HLA-C1 (*n*/%)	7 (15.5)	4 (14.81)	12 (30)	0.24
2DL3-HLA-C1 (*n*/%)	15 (33.3)	8 (29.62)	13 (32.5)	0.67
2DS2-HLA-C1 (*n*/%)	13 (28.87)	11 (40.74)	14 (35)	0.54
2DS2-HLA-C2 (*n*/%)	8 (17.77)	9 (33.3)	11 (27.5)	0.40
2DL2-HLA-C2 (*n*/%)	8 (17.77)	7 (25.92)	9 (22.5)	0.24
2DS1-HLA-C2 (*n*/%)	5 (11.11)	2 (7.40)	7 (17.5)	0.67
2DL1-HLA-C1 (*n*/%)	2 (4.4)	4 (14.81)	9 (22.5)	0.047
2DL2-HLA-C1 (*n*/%)	7 (15.5)	4 (14.81)	12 (30)	0.24
2DL1-HLA-C2 (*n*/%)	7 (15.5)	3 (11.11)	7 (17.5)	0.88

*KIR* killer cell immunoglobulin-like receptor, *HLA* human leukocyte antigen

**Fig. 2:**
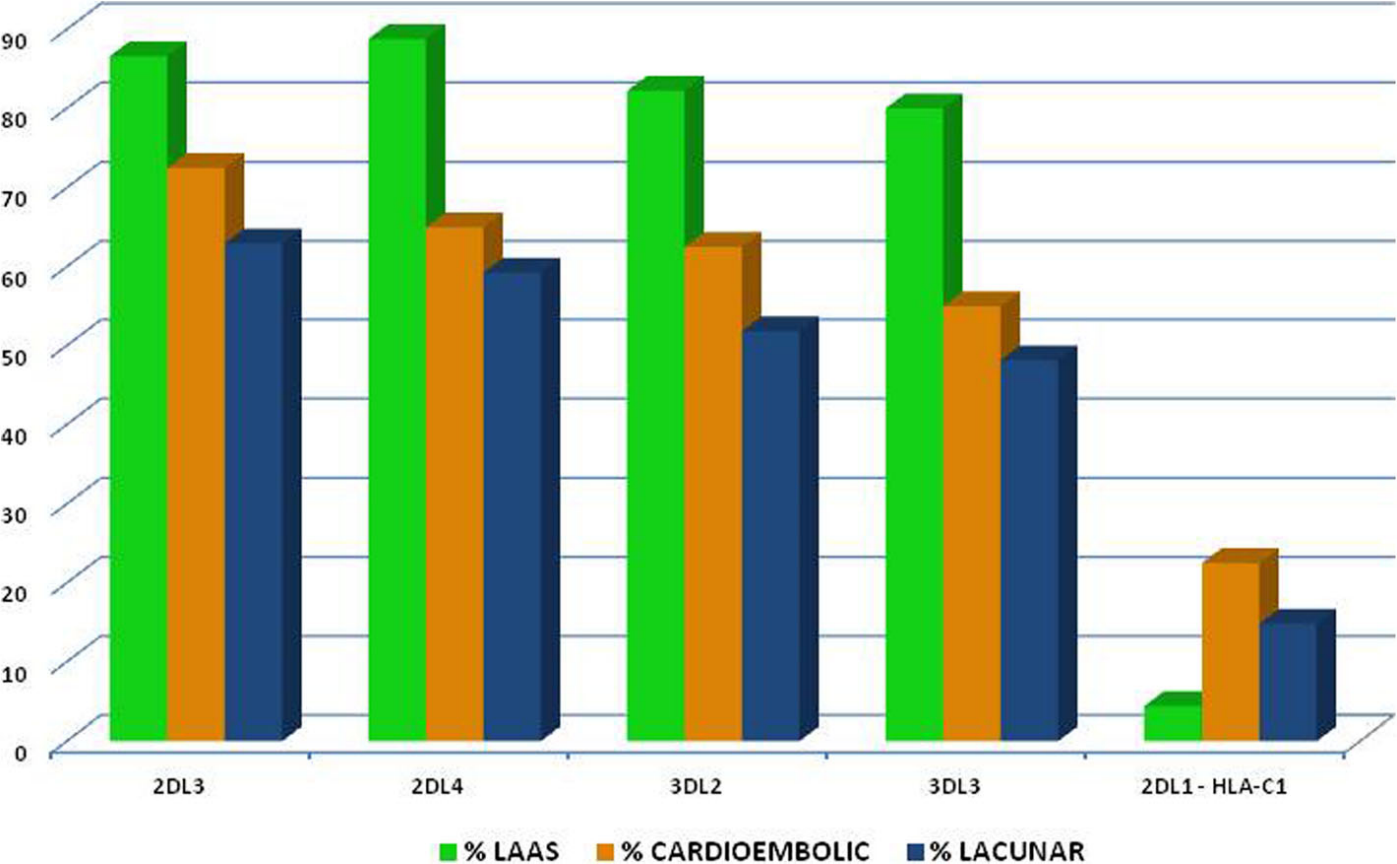
Frequency of KIR genes and of interaction KIR-HLA in subjects with ischemic stroke in relation to TOAST subtype

With regard to KIR gene frequency in relation to TOAST subtype, we observed a higher frequency of 2DL3 (86.3% vs 62.9% vs 72.5%; *p* = 0.033), 2DL4 (88.8% vs 59.25% vs 65%; *p* = 0.010), 3DL2 (82.2% vs 51.8% vs 62.5%; *p* = 0.016), and 3DL3 (80% vs 48.14% vs 55%; *p* = 0.015) KIR genes in subjects with LAAS subtype in comparison with subjects with other subtypes, whereas with regard to interactions between KIR and HLA alleles, we observed only a higher frequency of the interaction between KIR2DL1 and HLA-C1 alleles in subjects with cardioembolic stroke subtype (22.5% vs 4.4% vs 14.81%; *p* = 0.047) (see Table 5 and Fig. 2).

Multiple logistic regression analysis considering variables predictive of TOAST subtype of ischemic stroke showed a significant association between 2DL5A (*B* = 2.81; *p* = 0.03) and 3DL1 (*B* = 2.17; *p* = 0.02) KIR genes and cardioembolic stroke, whereas with regard to HLA alleles, only HLA-C1 allele (*B* = 2.36; *p* = 0.004) was significantly associated with cardioembolic stroke (see Tables 6 and 7).

**Table 6:** Logistic regression model to predict TOAST subtype of acute ischemic stroke in relation of prevalence of KIR haplotypes

Variable	Beta	Exp(B)	95% confidence interval for Exp(B)	*p* value
Lacunar subtype				
2DL1	0.176	1.193	0.32–4.43	0.79
2DL2	− 0.740	0.477	0.15–1.48	0.20
2DL3	− 0.588	0.555	0.12–2.52	0.44
2DL4	− 1.399	0.247	0.046–1.31	0.10
2DL5A	− 2.361	0.094	0.008–1.121	0.06
2DL5B	− 0.155	0.856	0.23–3.15	0.81
2DS1	− 0.035	0.965	0.21–4.25	0.96
2DS2	0.737	2.089	0.65–6.70	0.21
2DS3	− 0.477	0.620	0.17–2.16	0.45
2DS4	− 0.255	0.775	0.25–2.32	0.65
2DS5	0.273	1.314	0.30–5.58	0.71
3DL1	− 1.517	0.219	0.046–1.03	0.056
3DL2	0.913	2.491	0.34–18.27	0.36
3DL3	0.493	1.637	0.22–11.98	0.62
3DS1	0.105	1.110	0.33–3.70	0.86
2DP1	− 0.353	0.702	0.12–3.84	0.68
3DP1	− 1.022	0.360	0.063–2.07	0.25
3DP1003	− 0.123	0.884	0.10–7.21	0.90
LAAS subtype				
2DL1	− 0.24	0.786	0.18–3.46	0.751
2DL2	1.066	2.903	0.79–10.54	0.105
2DL3	0.91	2.486	0.33–18.84	0.378
2DL4	2.22	9.214	0.97–87.79	0.054
2DL5A	2.05	7.782	0.57–106.84	0.125
2DL5B	− 0.50	0.605	0.13–2.70	0.510
2DS1	− 0.68	0.502	0.09–2.80	0.433
2DS2	− 1.07	0.341	0.09–1.27	0.109
2DS3	1.24	3.464	0.79–15.19	0.100
2DS4	0.43	1.552	0.43–5.57	0.500
2DS5	− 1.16	0.312	0.048–2.03	0.224
3DL1	0.88	2.419	0.38–15.33	0.348
3DL2	− 1.031	0.357	0.02–4.45	0.424
3DL3	− 0.829	0.437	0.026–7.33	0.565
3DS1	− 0.244	0.783	0.19–3.09	0.727
2DP1	− 0.053	0.948	0.099–9.06	0.963
3DP1	0.771	2.161	0.20–22.5	0.519
3DP1003	1.096	2.993	0.21–43.26	0.421
Cardioembolic subtype				
2DL1	− 0.226	0.797	0.18–3.48	0.76
2DL2	0.144	1.155	0.31–4.28	0.83
2DL3	0.844	2.325	0.43–12.54	0.32
2DL4	0.801	2.227	0.35–14.04	0.39
2DL5A	2.81	16.672	1.23–225.4	0.03
2DL5B	0.657	1.928	0.45–8.21	0.37
2DS1	0.723	2.060	0.39–10.77	0.39
2DS2	− 0.264	0.768	0.21–2.83	0.69
2DS3	− 0.169	0.845	0.19–3.64	0.82
2DS4	0.058	1.060	0.31–3.65	0.92
2DS5	0.379	1.461	0.31–6.97	0.635
3DL1	2.17	8.791	1.41–54.6	0.02
3DL2	− 0.92	0.395	0.04–3.61	0.41
3DL3	− 0.88	0.413	0.04–4.06	0.44
3DS1	0.010	1.010	0.26–3.90	0.98
2DP1	0.75	2.119	0.31–14.59	0.44
3DP1	1.667	5.299	0.76–36.71	0.09
3DP1003	0.001	1.001	0.089–11.32	0.99

*LAAS* large artery atherosclerotic stroke, *KIR* killer cell immunoglobulin-like receptor, *HLA* human leukocyte antigen

**Table 7:** Logistic regression model to predict TOAST subtype of acute ischemic stroke in relation of prevalence of HLA allele and of HLA-KIR interactions

Variable	Beta	Exp(B)	95% confidence interval for Exp(B)	*p* value
Lacunar subtype				
HLA-A-Bw4	1.04	2.853	0.86–9.39	0.08
HLA-C1	− 1.24	0.28	0.07–1.15	0.07
HLA-C2	− 0.29	0.74	0.21–2.61	0.65
HLA-B-Bw4^T^	− 0.82	0.43	0.11–1.73	0.23
HLA-B-Bw4^I^	− 1.132	0.32	0.03–3.15	0.33
2DL2-HLA-C1	− 0.50	0.60	0.16–2.19	0.44
2DL3-HLA-C1	− 0.64	0.52	0.17–1.57	0.25
2DS2-HLA-C1	0.49	1.647	0.59–4.57	0.33
2DS-2HLA-C2	0.76	2.156	0.67–6.85	0.19
2DL2-HLA-C2	0.06	1.063	0.31–3.56	0.92
2DS1-HLA-C2	− 0.73	0.48	0.08–2.86	0.42
2DL1-HLA-C1	0.18	1.197	0.28–4.96	0.80
2DL1-HLA-C2	− 0.24	0.78	0.17–3.49	0.75
aplotipoHLA-A	− 0.38	0.68	0.25–1.79	0.44
LAAS subtype				
HLA-A-Bw4	− 1.322	0.26	0.07–1.01	0.05
AB haplotype	− 0.13	0.87	0.27–2.8	0.82
HLA-C1	0.31	1.37	0.29–6.36	0.68
HLA-C2	− 0.46	0.62	0.14–2.66	0.52
HLA-B-Bw4^T^	1.006	2.73	0.59–12.53	0.19
HLA-B-Bw4^I^	0.87	2.4	0.19–30.19	0.49
2DL2-HLA-C1	0.10	1.1	0.26–4.66	0.89
2DL3-HLA-C1	0.48	1.63	0.47–5.60	0.43
2DS-2HLA-C1	− 0.89	0.4	0.12–1.33	0.13
2DS2-HLA-C2	− 1.35	2.58	0.06–1.03	0.05
2DL2-HLA-C2	− 0.12	0.88	0.22–3.41	0.85
2DS1-HLA-C2	0.79	2.2	0.31–15.67	0.43
2DL1-HLA-C1	− 1.46	0.23	0.03–1.64	0.14
2DL1-HLA-C2	− 0.01	0.98	0.19–5.03	0.98
Cardioembolic subtype				
HLA-A-Bw4	− 0.48	0.61	0.15–2.40	0.48
AB-haplotype	− 1.18	0.3	0.08–1.15	0.08
HLA-C1	2.36	10.63	2.10–53.78	0.004
HLA-C2	1.24	3.48	0.71–17.07	0.12
HLA-B-Bw4^T^	0.96	2.63	0.51–13.38	0.24
HLAB-B-w4^I^	2.135	8.45	0.71–100.69	0.09
2DL2-HLA-C1	0.64	1.91	0.43–8.33	0.38
2DL3-HLA-C1	0.67	1.96	0.52–7.29	0.31
2DS2-HLA-C1	0.26	1.3	0.37–4.56	0.67
2DS2-HLA-C2	− 0.05	0.95	0.21–4.14	0.94
2DL2-HLA-C2	− 0.29	0.74	0.18–3.05	0.68
2DS1-HLA-C2	0.68	1.97	0.27–14.06	0.49
2DL1-HLA-C1	0.60	1.82	0.40–8.31	0.43
2DL1-HLA-C2	0.78	2.19	0.39–12.19	0.36

*LAAS* large artery atherosclerotic stroke, *KIR* killer cell immunoglobulin-like receptor, *HLA* human leukocyte antigen

At multiple regression analysis, we also observed a possible trend toward significance with regard to the protective role of HLA-A-Bw4 HLA alleles (*p* = 0.05) and of interaction between KIR 2DS2 and HLA-C2 alleles (*p* = 0.05) towards LAAS subtype of stroke (see Table 7).

## Discussion

Our study showed that subjects with acute ischemic stroke in comparison with subjects without stroke were more likely to have a higher frequency of 2DL3, 2DL4, 2DL5B, 2DS2, 2DS4, and 3DP1 KIR genes and a significantly lower frequency of HLA-B-Bw4^I^ allele.

The definition of an immunological genetic profile in patients with acute ischemic stroke could better characterize the pathogenesis of the disease, offering new perspectives of diagnostic and therapeutic evaluation. Considering the complexity of genetic studies, we hypothesized that due to the high polymorphism of KIR genes by contributing to the variability of the immune response both innate and adaptive, these genes could be a suitable subject of a study addressed to explain genetic basis of inflammatory pathogenesis of ischemic stroke and of its subtype.

Several studies have reported that susceptibility to ischemic stroke and its functional prognosis seems to be influenced by systemic inflammatory processes and that stroke patients with systemic inflammation exhibit clinically poorer outcomes [14].

The KIRs (also known as CD158) are a family of receptors encoded by 14 polymorphic genes [15] seven of which are inhibitory and seven of which are activating. Although the current view focuses primarily on the impacts of KIR expression on NK cell function, it is important to note that KIR are also expressed on subsets of T cells, including invariant NKT cells, and can also directly influence their function.

Furthermore, some authors [16–19] have shown that the relative response of CD56^bright^ and CD56^dim^ NK cells is associated with KIR genotype, providing the first direct evidence that KIR genotype may influence the NK response to a wide range of non-viral pathogens.

In our study, we reported higher serum levels of IL-6 and TNF-α and higher percentage of peripheral CD4+ cells and CD4+CD28 null in subjects with acute ischemic stroke in comparison with subjects without stroke. These findings are consistent with our own previous results [27, 33, 34] and with other previous studies reporting the role of an immune-inflammatory activation involving inflammatory cytokines [20, 21] and CD4+CD28 null T cell subsets [22–25] in the acute phase of ischemic stroke. Thus, our finding of an increased peripheral percentage of CD28^null^ cells in peripheral blood of subjects with ischemic stroke may be also related to the higher prevalence of KIR gene activators such as 2S2 and 2DS4.

CD4^+^CD28^null^ T cells are functionally distinct from classical CD4 helper T cells. They do not express CD40 ligand, produce large amounts of IFN-γ, and express granzyme B and perforin, giving them the capability to lyse target cells [30, 31]. Their cytotoxic potential, as well as expression of the NK cell marker CD57, suggests that CD4^+^CD28^null^ T cells share features with NK cells. CD4^+^CD28^null^ but not CD4^+^CD28^+^ T cells express KIR.

A direct role of CD4^+^CD28^null^ T cells in vascular injury has been suggested because these cells are also a risk factor for inflammation and rupturing of atherosclerotic plaques in acute coronary syndromes [32].

These findings corroborate an inflammatory conception of ischemic stroke, although since inflammation has a validated role in plaque instability events, inflammatory mechanisms may explain atherothromboembolic pathogenetic mechanisms of stroke.

In a previous study [32], we postulated that a potential mechanism for the relationship between inflammation, stroke pathogenesis, and stroke subtype pathogenesis may be focused on the contribution of pro-inflammatory gene polymorphisms (SNPs).

In our subjects with acute ischemic stroke in comparison with control subjects, we observed a higher prevalence of certain activators KIR gene (2DS2 and 2DS4) consistent with findings of previous studies conducted in patients with acute coronary syndrome [26–28] and unstable atherosclerotic plaques [29]. We have also reported a higher prevalence of an inhibitor gene such as 2DL3, of one aplotype of uncertain behaviour such as KIR2DL5B, and of one gene such as KIR2DL4 that is both inhibitory and activating signaling. It is conceivable to consider the prevailing KIR gene frequency of patients with acute ischemic stroke as predominantly as excitatory consistently with previous findings in patients with coronary acute syndrome, whereas coexistent expression of the two inhibitor genes could be viewed as a simple modulatory effect towards excitatory pathway expression.

The function of stimulatory KIRs is unclear, and these receptors are usually expressed in the context of several inhibitory receptors. For the CD4^+^CD28^null^ T cell clones, some authors [33] recently demonstrated a costimulatory activity of CD158b-specific antibodies to enhance proliferation and cytokine production [34], confirming earlier reports that KIR2DS2 can function as a costimulatory molecule in response to suboptimal TCR triggering [35]. Furthermore, it has been reported in patients with rheumatoid vasculitis [36] and in patients with an acute coronary syndrome [37, 38] that CD4^+^CD28^null^ T cells expressed KIR2DS2 in the absence of opposing inhibitory receptors of the same specificity. Thus, in CD4^+^CD28^null^ T cells, the activity of KIR2DS2 may favor the activation of autoreactive T cells involved in instability mechanisms of atherosclerotic plaque and in ischemic neuronal damage.

With regard to HLA alleles, our patients with acute ischemic stroke showed in comparison with subjects without stroke no significant difference in frequency of HLA alleles, whereas subjects without ischemic stroke showed in comparison with stroke subjects a significantly higher frequency of HLA-B-Bw4^I^ allele. The increased expression in control subjects without ischemic stroke of the HLA-B with “Bw4^I^ motif” that generally interacts with inhibitory 3DL1 and 3DL3 receptors could represent a further confirmation of predominantly pro-inflammatory genetic background of subjects with stroke in comparison with the anti-inflammatory genetic profile of control subjects without ischemic stroke. To our knowledge, this is the first study to show that the KIR-ligand group HLA-A-Bw4^I^ can influence the susceptibility to ischemic stroke and may also represent a possible pathogenetic background of inflammatory damage in acute ischemic stroke. This effect may be mediated by the activation of the inhibitory KIR3DL1, although an association with other HLA-B-Bw4 alleles [37, 38] that bind the same KIR gene was not reported in this study.

Finally, multiple logistic regression analysis considering variables predictive towards the presence of acute ischemic stroke showed a protective effect of HLA-B-Bw4^I^ and of 2DL2-HLA-C1, and a detrimental effect of 2DL2-HLA-C1_A and 2DS2-HLA-C2 HLA-KIR interactions.

These findings could confirm the protective role of an anti-inflammatory genetic background such as the presence of the B-Bw4^I^ motif.

Thus, our present study represents a first attempt to bring in the context of an overall immune-inflammatory view of point that leads cytokine and cell-dependent inflammatory mechanisms ischemic stroke occurrence to a genetic background in terms of KIR receptor-ligand HLA allele interactions. Thus, a greater degree of cytokine and cell-mediated (NK cells or T cell subset such as CD4^+^CD28^nul^l cells) inflammatory activation could be also dependent by KIR genes responsible for activator KIR receptors on these inflammatory cells.

Further research on other studies by our group will be needed, and some studies are already been addressed to evaluate possible effects on the incidence of recurrent ischemic events in relation to frequency of KIR and HLA genes in patients with ischemic stroke.

With regard to KIR gene frequency in relation to TOAST subtype, we observed a higher frequency in subjects with LAAS subtype in comparison with subjects with other TOAST subtypes of 2DL3, 2DL4, 3DL2, 3DL3, and 3DP1*003 KIR genes, whereas with regard to interaction KIR-HLA, we observed a higher frequency of 2DL1HLAC1 in subjects with cardioembolic stroke subtype.

Multiple logistic regression analysis considering variables predictive of TOAST ischemic stroke showed a significant association between 2DL5A and 3DL1 KIR genes and cardioembolic stroke, whereas with regard to HLA allele interaction, only HLA-C1 allele was significantly associated to cardioembolic stroke. We also observed a possible trend toward significance with regard to a possible protective role of HLA-A-Bw4 HLA allele and of 2DS2-HLA-C2 interaction HLA-KIR towards LAAS subtype of stroke.

These findings seem to be not fully consistent with the findings of this study of a prevailing KIR gene frequency of patients with acute ischemic stroke as predominantly as excitatory, whereas the analysis between TOAST subtypes such as atherosclerotic and cardioembolic ones shows an association with prevailing inhibitory genes such as 2DL3, 2DL4, 3DL2, and 3DL3 KIR genes and a prevailing inhibitory HLA-KIR interaction such as 2DL1HLAC1.

It explains that the relationship between KIR genes and stroke subtypes is not easy to explain, and probably future studies with a larger sample of subjects with each stroke subtype may be helpful to better characterize the relationship between HLA-KIR genetic background and clinical subtype of ischemic stroke.

Nevertheless, some possible explanations may be postulated to explain the non-consistent findings of our two sets of analysis with regard to the association between KIR and HLA genes with stroke and stroke subtypes.

Several evidences support the concept that migration of inflammatory cells to the vascular wall is intimately associated with the cause of vascular conversion leading to atherosclerosis [39–41]. Inflammatory cells in cerebral vessel have been reported as capable of responding to cardiovascular risk factors for human stroke such as hypertension, hyperlipidemia, diabetes mellitus, obesity, and smoking in subjects with cerebrovascular disease. The role of inflammatory cell in stroke pathogenesis has recently been reported, but the role of KIR-HLA interaction to regulate migration and response of inflammatory cells such as NKs and T cell in development of a cerebral artery thrombosis due to atherosclerotic and cardioembolic mechanism is unknown.

HLA class I molecules and the KIR serve as ligand and receptor. Certain combinations of KIRs and HLA molecules can transmit activating or inhibitory signals to regulate the function of NK cells.

HLA-C alleles can be placed into one of two categories, C1 and C2, which are recognized differentially by the KIR receptors. NK cells that express KIR2DL2 and KIR2DL3 interact with HLA-C1 allotypes, whereas KIR2DL1 and KIR2DS1 interact with HLA-C2 allotypes [42–46]. Our findings of a higher prevalence of HLA-C1 HLA alleles and of 3DL1 KIR gene in subjects with cardioembolic stroke could underline the prevalent role of inhibitory mechanisms of NKs and T cell regulating, whereas an excitatory pathway seems to be more involved in subjects with atherosclerotic strokes.

Nevertheless, recent reports indicate that the interaction of the inhibitory KIR with the HLA ligand not only functions to inhibit NK cell activation, but also serves to prime the particular NK cell for activation in the absence of self [47, 48].

An individual NK or T-lymphocyte cell may simultaneously express multiple different receptors, each of which may have different ligand specificities. Furthermore, in addition to the known inhibitory role of KIR at the effector level, coinheritance of inhibitory KIR2DL3 and its cognate ligand HLA-CAsn-80 is associated with increased clearance of hepatitis C virus [49]. These findings indicate that in addition to their inhibitory function at the effector level, another critical role for these MHC-specific inhibitory receptors was recently described [50], a process that these authors have termed licensing and that involves a positive role for MHC-specific inhibitory receptors and requires the cytoplasmic inhibitory motif originally identified in effector responses after interactions of inhibiting KIR receptor with its ligand thus resulting in an immunoinflammatory activation. This issue could also explain our findings of association between inhibitory KIR genes and inhibitory KIR-HLA interactions in subjects with atherosclerotic subtype of stroke.

Licensing process of NKs and T cells may probably help to explain different effects of KIR and HLA repertoire in different subtype of strokes but future studies addressed to analyze these differences in larger sample of subjects with acute ischemic stroke may contribute to add further advances about this issue.

### Limitations

The main limitation of this study lies in its cross-sectional nature, making it impossible to dissect the temporal relation between genetic background and progression of cerebrovascular disease.

Another limitation is due to the fact that CD4+CD28^null^ T cells variably express receptors of the killer immunoglobulin-like receptor (KIR) family; nevertheless, we do not perform T cell cloning and KIR phenotyping of T cell clones on total RNA extracted from T cell clone with an evaluation of different KIR gene transcripts and the encoded proteins as CD158 [51].

## Conclusions

Our study showed that subjects with acute ischemic stroke in comparison with subjects without stroke were more likely to have a higher frequency of 2DL3, 2DL4, 2DL5B, 2DS2, 2DS4, and 3DP1 KIR genes and a significantly lower frequency of HLA-B-Bw4^I^ allele. It explains that the prevailing KIR phenotype of patients with acute ischemic stroke is predominantly as excitatory consistently with previous findings in patients with coronary acute syndrome. Our findings concerning KIR genotyping and evaluation of peripheral percentage of CD4+ and CD28^null^ cells in subjects with acute ischemic stroke may offer useful information in order to better understand genetic determinants of inflammatory pathogenesis of ischemic stroke and to offer possible future prognostic and therapeutic tools.

## Abbreviations

24 hour ECG: 24 h electrocardiography monitoring
2DL3: 2DL3 killer cell immunoglobulin-like receptor gene
2DL5B: 2DL5B killer cell immunoglobulin-like receptor gene
2DS2: 2DS2 killer cell immunoglobulin-like receptor gene
ACS: Acute coronary syndrome
AMI: Acute myocardial infarction
CD158j: Human natural killer cell activating receptor KIR2DS2
CD4: Cluster of differentiation 4
CD8: Cluster of differentiation 8
CEI: Cardioembolic infarct
CT: Computerized tomography
CU: Carotid ultrasound
DAP12: DNAX activation protein of 12 kDa
ECG: Electrocardiogram
ESH/ESC: European Society of Hypertension/ European Society of Cardiology
HLA: Human leukocyte antigen
HLA-A: Human leukocyte antigen-B
HLA-B: Human leukocyte antigen-B
HLA-B-Bw4^I^: Human leukocyte antigen–B-Bw4 motif I allele
HLA-Bw4: Human leukocyte antigen-Bw4
HLABw4^T^: Human leukocyte antigen–B-Bw4 motif T allele
HLA-C1: Human leukocyte antigen-C1
HLA-C2: Human leukocyte antigen-C2
IgG1-FITC: IgG1-fluorescein isothiocyanate
IgG2a-PE: Immunoglobulin gG2a conjugated with phycoerythrine
KIRIIND: KIR Infectious and Inflammatory Disease Collaborative Group
KIRs: Killer cell immunoglobulin-like receptor
LAAS: Large artery atherosclerosis
LAC: Lacunar infarct
MRI: Magnetic resonance imaging
NCEP/ATP III: National Cholesterol Education Program–Adult Treatment Panel III
NK cells: Natural killer cells
NK: Natural killers
ODE: Stroke of other determined etiology
PBMCS: Peripheral blood mononuclear cells
T_H_1: T-helper type 1
TIA: Transient ischemic attack
TOAST: Trial Org 10172 in acute stroke treatment
TTE: Trans-thoracic echocardiography
UDE: Stroke of undetermined Etiology
